# Alpha synuclein post translational modifications: potential targets for Parkinson’s disease therapy?

**DOI:** 10.3389/fnmol.2023.1197853

**Published:** 2023-05-25

**Authors:** Viviana Brembati, Gaia Faustini, Francesca Longhena, Arianna Bellucci

**Affiliations:** Division of Pharmacology, Department of Molecular and Translational Medicine, University of Brescia, Brescia, Italy

**Keywords:** alpha synuclein, post translational modifications, Parkinson’s disease, synucleinopathies, therapeutic targets, biomarkers

## Abstract

Parkinson’s disease (PD) is the most common neurodegenerative disorder with motor symptoms. The neuropathological alterations characterizing the brain of patients with PD include the loss of dopaminergic neurons of the nigrostriatal system and the presence of Lewy bodies (LB), intraneuronal inclusions that are mainly composed of alpha-synuclein (α-Syn) fibrils. The accumulation of α-Syn in insoluble aggregates is a main neuropathological feature in PD and in other neurodegenerative diseases, including LB dementia (LBD) and multiple system atrophy (MSA), which are therefore defined as synucleinopathies. Compelling evidence supports that α-Syn post translational modifications (PTMs) such as phosphorylation, nitration, acetylation, O-GlcNAcylation, glycation, SUMOylation, ubiquitination and C-terminal cleavage, play important roles in the modulation α-Syn aggregation, solubility, turnover and membrane binding. In particular, PTMs can impact on α-Syn conformational state, thus supporting that their modulation can in turn affect α-Syn aggregation and its ability to seed further soluble α-Syn fibrillation. This review focuses on the importance of α-Syn PTMs in PD pathophysiology but also aims at highlighting their general relevance as possible biomarkers and, more importantly, as innovative therapeutic targets for synucleinopathies. In addition, we call attention to the multiple challenges that we still need to face to enable the development of novel therapeutic approaches modulating α-Syn PTMs.

## 1. Introduction

Parkinson’s disease (PD) is the second most common movement disorder, affecting 2% of the world population over 65 years of age (Baker and Graham, 2004).

Motor symptoms mainly arise from the loss of dopaminergic nigrostriatal neurons, that alters the homeostasis of basal ganglia networks (Hornykiewicz, 2001). Beyond motor manifestations, PD patients may also exhibit a wide range of non-motor and psychiatric symptoms, which are caused by functional changes in central nervous system (CNS) and peripheral network system (PNS) circuits (Pfeiffer, 2016; Engelender and Isacson, 2017; Takamatsu et al., 2018; Kulkarni et al., 2022).

Key neuropathological hallmark of PD is the deposition of insoluble proteinaceous inclusions in cell bodies and neurites (Gibb, 1986), which are called Lewy bodies (LB) and Lewy neurites (LN), respectively. In 1997, these were found to be mainly composed of alpha synuclein (α-Syn) insoluble fibrils (Spillantini et al., 1997). In the last decades, it has been shown that α-Syn is particularly enriched at synaptic terminals, where it regulates synaptic function (Spillantini et al., 1997; Burre et al., 2010; Longhena et al., 2019). Since then, other disorders such as LB dementia (LBD), multiple system atrophy (MSA), Alzheimer’s disease (AD) LB variant or neurodegeneration with brain iron accumulation (NBIA), have been found to be characterized by brain accumulation of insoluble α-Syn deposits, and have been defined as synucleinopathies (Spillantini et al., 1998; Spillantini, 1999; Spillantini and Goedert, 2016; Goedert et al., 2017).

Interestingly, α-Syn deposits have been observed also in the PNS innervating the gastrointestinal tract, blood, salivary glands, olfactory mucosa, skin, retina, adrenal gland, heart and muscles (Qualman et al., 1984; Fumimura et al., 2007; Beach et al., 2010; Gelpi et al., 2014; Zange et al., 2015; Stoessl, 2016; Rey et al., 2016a,b, 2018; Wakabayashi, 2020). This peripheral α-Syn pathology is thought to contribute to the onset of PD non-motor manifestations in the prodromal and symptomatic phase (Abbott et al., 2007).

Remarkably, numerous studies in experimental models of synucleinopathy, post-mortem PD brains and neuroimaging evidences support that α-Syn pathological aggregation can severely impair synaptic function, thus consequently perturbing neuronal network dynamics and inducing neurodegeneration (Bellucci et al., 2016, 2017; Longhena et al., 2017, 2019; Kulkarni et al., 2022). This notwithstanding, we still ignore the mechanisms that drive pathological α-Syn aggregation in neuronal cells, and this has hampered the development of innovative effective therapies that block α-Syn pathological deposition as disease modifying approaches for PD and other synucleinopathies (Fields et al., 2019; Lashuel, 2021; Oliveira et al., 2021; Engelender et al., 2022). Indeed, current α-Syn-targeting strategies mainly include immunotherapy-based removal of extracellular α-Syn fibrils, gene therapy-based reduction of α-Syn, general and non-selective small molecule inhibitors of protein aggregation and protein degradation enhancers, but we still miss a cutting edge approach interfering with the culprit of α-Syn aggregate formation.

Interestingly, several post translational modifications (PTMs) of α-Syn have been found to differently modulate α-Syn aggregation either by predisposing or interfering with it (Zhang et al., 2019; Table 1). Indeed, they can affect α-Syn aggregation propensity, solubility and turnover, membrane binding and interaction with other proteins and metals (Oueslati et al., 2010; Zhang et al., 2017, 2019; Bell and Vendruscolo, 2021; Bell et al., 2022a,b). Moreover, α-Syn PTMs can serve as markers for environmental changes, may play a role in gene expression by impinging on cellular responses to stimuli and are also under study as possible disease biomarkers for synucleinopathies (Vicente Miranda et al., 2017a; Fayyad et al., 2019; Vivacqua et al., 2019; Petricca et al., 2022; Sonustun et al., 2022).

**Table 1:** Functional consequences of the majorly studied α-Syn PTMs.

PTMs	Amino acid	Effects	References
Phoshorylation	ser87	Limits aggregation	Paleologou et al. (2010)
	ser129	Promotes aggregation	Fujiwara et al. (2002), Smith et al. (2005b), Karampetsou et al. (2017)
		Limits aggregation	Gorbatyuk et al. (2008), Oueslati et al. (2012), Tenreiro et al. (2014), Ghanem et al. (2022)
	tyr39	Promotes aggregation	Brahmachari et al. (2016), Mahul-Mellier et al. (2014), Dikiy et al. (2016)
	tyr125	Limits aggregation	Negro et al. (2002), Chen et al. (2009), Kosten et al. (2014)
Nitration	tyr39	Promotes oligomerization	Hodara et al. (2004)
	tyr125	Promotes dimerization	Takahashi et al. (2002), Hodara et al. (2004)
	tyr133	Promotes aggregation	Hodara et al. (2004)
	tyr136	Promotes aggregation	Hodara et al. (2004)
N-terminal acetylation		Inhibits aggregation	Bell et al. (2022a,b, 2023)
O-GlcNAcylation	thr72	Inhibits aggregation	Marotta et al. (2015), Levine et al. (2017, 2019)
	ser87	Inhibits aggregation	Lewis et al. (2017)
Glycation	lys	Promotes oligomerization	Vicente Miranda et al. (2017b)
SUMOylation	lys	Limits aggregation	Krumova et al. (2011)
	lys	Protects from degradation	Rott et al. (2017), Rousseaux et al. (2018)
Ubiquitination	lys	Promotes degradation	Tofaris et al. (2011), Engelender et al. (2022)

In this review, we summarize and discuss the main findings on α-Syn PTMs, in order to define a route to decipher whether these modifications can be rationally considered as achievable druggable targets for synucleinopathies or effective biomarkers monitoring the progression or enabling patient stratification in these neurodegenerative disorders.

## 2. α-Syn and its post-translational modifications

α-Syn is a member of synuclein family, which also includes β-and γ-synuclein (Clayton and George, 1998). In humans, α-Syn is encoded by the SNCA gene located on chromosome 4q21 (Shibasaki et al., 1995; Lavedan, 1998).

Although the physiological role of α-Syn has not been fully elucidated yet, numerous studies demonstrated its involvement in the control of synaptic release. Indeed, it regulates synaptic vesicle clustering, the coupling and fusion of vesicles participating in SNARE complex assembly, the extent of phasic and tonic neurotransmitter release as well as neurotransmitter reuptake (Choi et al., 2013; Ghiglieri et al., 2018; Longhena et al., 2019). Moreover, α-Syn regulates mitochondrial function, fusion as well as mitochondria and endoplasmic reticulum interaction at mitochondria-associated membranes (MAM; Dauer et al., 2002; Ellis et al., 2005; Di Maio et al., 2016; Ludtmann et al., 2016; Menges et al., 2017; Faustini et al., 2019; Risiglione et al., 2021; Thorne and Tumbarello, 2022) and is involved in neuronal plasticity (Liu et al., 2004b, 2007; Watson et al., 2009; Ullman et al., 2011; Leite et al., 2022; Calabresi et al., 2023).

α-Syn is composed of 140 amino acids and its molecular weight is 14 kDa. α-Syn structure encompasses 3 domains: (1) the N-terminal region (residues 1–60), is positively charged and contains imperfect repeats with a highly conserved hexameric motif (KTKEGV), typically involved in the formation of amphipathic α-helices which mediate membrane binding (Clayton and George, 1998; George, 2002; Vamvaca et al., 2009); (2) the central hydrophobic region (residues 61–95), also known as non-amyloid component (NAC) portion, is prone to intermolecular interactions and is crucial for aggregation and fibril formation (Giasson et al., 2001; Ma et al., 2003); (3) the C-terminal region (residues 96–140) is highly enriched in acidic proline residues (Bellucci et al., 2012). This part of the protein reduces the NAC propensity for aggregation, mediates the majority of α-Syn interactions with proteins, metal ions and other ligands, including dopamine and polyamines, and harbors the majority of PTMs sites (Jensen et al., 1999; Paik et al., 1999; Giasson et al., 2003; Fernandez et al., 2004; Hoyer et al., 2004; Brown, 2007).

α-Syn does not present a defined structure in aqueous solutions and for this reason is defined “natively unfolded” (Stefanis, 2012), but it can shift to α-helix structure in association with membrane phospholipids, suggesting that it acquires different roles in different subcellular compartments based on its dynamic structure (Ahn et al., 2002). Indeed, in function of its capacity to acquire different conformations, α-Syn can interact with lipid membranes, enzymes, chaperones, synaptic and cytoskeletal proteins. Some studies also suggested a physiological α-helical structure forming dimers that counteract synaptic vesicle fission or tetramers that resist aggregation (Bartels et al., 2011; Wang et al., 2011; Medeiros et al., 2017).

Compelling evidence supports that PTMs play an important role in promoting conformational changes that make α-Syn more or less prone to aggregation (Table 1). Indeed, several PTMs such as phosphorylation, nitration, acetylation, glycation, truncation, ubiquitination, SUMOylation and O-GlcNAcylation can affect α-Syn structure. In particular, PTMs can either promote or inhibit α-Syn oligomerization, fibrillization and degradation (Feany and Bender, 2000; Fujiwara et al., 2002; Hodara et al., 2004; Smith et al., 2005a; Kasai et al., 2008; Lee et al., 2008; Rott et al., 2008, 2017; Tetzlaff et al., 2008; Danielson et al., 2009; Oueslati et al., 2010, 2013; Levine et al., 2017; Lewis et al., 2017; Zhang et al., 2019). Moreover, it has been described that LB contain phosphorylated, nitrated, ubiquitinated, SUMOylated and C-terminally truncated α-Syn, further supporting the role of PTMs in the modulation of α-Syn aggregation (Baba et al., 1998; Crowther et al., 1998; Giasson et al., 2000; Gomez-Tortosa et al., 2000; Campbell et al., 2001; Hasegawa et al., 2002; Anderson et al., 2006; Paleologou et al., 2010; Rott et al., 2017).

## 3. α-Syn post-translational modifications as possible biomarkers for PD and other synucleinopathies

Of note, α-Syn and post translational modified α-Syn in peripheral and accessible tissues have been investigated as possible biomarkers for the diagnosis of PD and other synucleinopathies. Nevertheless, since none of them has been validated across different cohorts so far, we still miss a clear cut evidence supporting their factual clinical significance (Witt et al., 2009; Pouclet et al., 2012; Shannon et al., 2012; Donadio et al., 2014, 2018; Sprenger et al., 2015; Zange et al., 2015; Stokholm et al., 2016; Vilas et al., 2016; Fereshtehnejad et al., 2017).

Biomarkers are defined as cellular, biochemical or molecular alterations that are measurable in biological samples such as human tissues, cells, or fluids (Hulka, 1990). The definition has been extended in order to define biomarkers as biological characteristics that can be objectively measured and evaluated as an indicator of normal biological processes, pathogenic processes, or pharmacological responses to a therapeutic intervention (Naylor, 2003). In particular, biomarkers include tools and technologies that can help disease prediction, cause, diagnosis, progression, regression, or the outcome of treatments (Mayeux, 2004). The importance of biomarkers is particularly relevant in the context of diseases affecting CNS, where it is impossible to have the direct access to the unhealthy tissue. CNS biomarkers detection can be pursued by positron emission tomography (PET) or magnetic resonance imaging (MRI) as well as by biological fluids [blood, cerebrospinal fluid (CSF), saliva], skin and gastrointestinal system biopsies or nasal mucosa analysis.

The fact that α-Syn can be found in different forms (monomeric, oligomeric, aggregated or post translational modified) in accessible and peripheral tissues such as CSF, blood, saliva, tears, colon, esophagus and skin (Tokuda et al., 2010; Devic et al., 2011; Foulds et al., 2011, 2012; Mollenhauer et al., 2013; Abd-Elhadi et al., 2015; Koehler et al., 2015; Chung et al., 2016; Cariulo et al., 2019; Fenyi et al., 2019; Hamm-Alvarez et al., 2019; Vivacqua et al., 2019; Maass et al., 2020; Wang et al., 2020b; Tanei et al., 2021; Bakhit et al., 2022), opened up the possibility to evaluate whether these different proteoforms may be useful for the diagnosis of PD and or other synucleinopathies (Witt et al., 2009; Pouclet et al., 2012; Shannon et al., 2012; Donadio et al., 2014, 2018; Sprenger et al., 2015; Zange et al., 2015; Stokholm et al., 2016; Vilas et al., 2016; Fereshtehnejad et al., 2017; Fayyad et al., 2019; Parnetti et al., 2019; Vivacqua et al., 2019, 2023; Wang et al., 2020b; Ganguly et al., 2021).

Several studies demonstrated that the levels of α-Syn phosphorylated at serine 129 (p-ser129), a PTM that is considered a marker of mature α-Syn aggregates (Ghanem et al., 2022), are elevated in the CSF and plasma of PD patients (Foulds et al., 2011, 2012; Wang et al., 2014; Landeck et al., 2016; Majbour et al., 2016a,b), while total α-Syn levels are decreased (Vivacqua et al., 2019, 2023). Remarkably, the levels of p-ser129 α-Syn were also found to significantly correlate with symptom severity in PD patients, suggesting that p-ser129 may serve as a biomarker for disease progression (Wang et al., 2014; Stewart et al., 2015).

In a recent study, increased levels of total and aggregated α-Syn in the membrane fraction of erythrocytes and high levels of p-ser129 α-Syn in cytosolic fractions were detected in PD cases versus healthy controls (Tian et al., 2019). Another report that analyzed oxidized and p-ser129 α-Syn demonstrated that higher levels of total and proteinase K resistant α-Syn and p-ser129 α-Syn can be detected in PD patients with motor symptoms (without dementia) with a high degree of accuracy (Abd Elhadi et al., 2019). Interestingly, p-ser129 α-Syn can be detected in skin nerve fibers biopsies and saliva (Vivacqua et al., 2019, 2023; Bougea et al., 2019a; Infante et al., 2020; Wang et al., 2020a; De Bartolo et al., 2023). Interestingly, α-Syn isolated from the skin and saliva has aggregation seeding activity and could serve as a biomarker for PD and as a differential biomarker to distinguish synucleoinopathies from tauopathies (Wang et al., 2020b).

p-ser129 α-Syn has also been detected in the lysate of red blood cells in synucleinopathies (Tian et al., 2019; Li et al., 2020, 2021). Higher levels of both Tyrosine (tyr) 125-phosphorylated α-Syn (p-tyr125) and p-ser129 α-Syn can be also detected in the blood of PD patients (Foulds et al., 2011, 2013; Vicente Miranda et al., 2017a).

Two recent meta-analysis showed that patients with PD have higher blood oxidative stress (OS) markers such as malondialdehyde (MDA), 8-Oxo-2′-deoxyguanosine lipid hydro-peroxide, nitrate and ferritine and lower antioxidant activity of superoxide dismutase (SOD), glucose 6 phosphate dehydrogenase, catalase, and glutathione peroxidase (GPx) compared with healthy control (Khan and Ali, 2018). Nitration of tyr and tryptophan residues as a consequence of the formation of peroxynitrite byproducts easily occurs at OS sites, i.e., in inflamed tissue, and can alter the structure and function of proteins. Nitric oxide (NO) and superoxide react to form peroxynitrite which promotes the nitrification of tyr residues in proteins. Specifically, the nitro group (−NO_2_) is added to replace a hydrogen atom in the 3′ position of the tyr phenolic ring to form 3-nitrotyrosine (Chavarria and Souza, 2013). Several studies reported the presence of nitrated α-Syn in *in vivo* and *in vitro* experimental models of PD and also in LB (Giasson et al., 2000; Yu et al., 2010; He et al., 2019; Manzanza et al., 2021; Simon et al., 2021; Magalhaes and Lashuel, 2022). Of note, Fernandez et al. (2013) reported the presence of tyr125/136 nitrated α-Syn in the CSF and serum of early PD patients, while a more recent study showed increased levels of nitrated α-Syn at tyr39 (n-tyr39) in the red blood cells of PD patients (Vicente Miranda et al., 2017a). In the same study, Vicente Miranda et al. (2017a) showed also reduced levels of SUMOylated α-Syn and increased levels of glycated α-Syn in PD patients erythrocytes with respect to controls. Since SUMOylation can increase α-Syn solubility and reduce aggregation (Krumova et al., 2011) and glycation can potentiate neuronal loss and motor impairment (Vicente Miranda et al., 2017b), the observed results may reflect brain α-Syn pathological alterations and toxicity (Vicente Miranda et al., 2017a,b).

These findings suggest that α-Syn PTMs, and in particular α-Syn nitration or phosphorylation, can be valuable biomarkers for synucleinopathies. This notwithstanding, we miss large cross-sectional and follow-up studies that will be pivotal for the implementation of post-translationally-modified α-Syn as a biomarker and we need to standardize the most reliable detection methods and several technical issues dealing with the detection or quantification of α-Syn have to be solved (Schmid et al., 2013; Mollenhauer et al., 2017; Magalhaes and Lashuel, 2022; Petricca et al., 2022). Indeed, the assay developed in the different studies exhibited different sensitivity and specificity and also led to conflicting results (Malek et al., 2014; Vivacqua et al., 2019, 2023; Bougea et al., 2019a,b; De Bartolo et al., 2023). For instance, Lin et al. (2019) recently reported a marked increase in total and phosphorylated α-Syn levels as well as in their ratio in the plasma of PD patients vs. healthy controls with assays exhibiting elevated specificity (AUC of ROC curves: 0.94, 0.91 and 0.74, respectively). This is in contrast to the findings of a previous study (Foulds et al., 2012) describing a reduction of total α-Syn and a parallel increase in phosphorylated α-Syn levels detected in the plasma of PD patients with a phosphorylated α-Syn assay exhibiting a ROC AUC = 0.68. Consistently, other reports showed that levels of phosphorylated α-Syn are increased in spite of the decrease of total α-Syn levels in plasma of PD patients (Hong et al., 2010; Gorostidi et al., 2012; Cariulo et al., 2019). When considering that because of sensitivity and specificity issues even CSF or plasma α-Syn cannot be considered as valuable markers of PD yet, it is clear that, as the reliable detection of post-translationally modified α-Syn is even more problematic, much work is warranted for achieving the exhaustive clinical translation of these kind of assay. This notwithstanding, the integrated measurement of α-Syn PTM may offer the possibility to single out patient-specific signatures that in the future could be of great help to settle precision-medicine-based approaches if disease-modifying therapies targeting α-Syn pathology will be developed.

## 4. Phosphorylation

Among α-Syn PTMs, phosphorylation is the most studied. The primary cause of this interest is mainly due to the fact that in normal brains only 4% of α-Syn is phosphorylated, whereas in LB extracted from PD brains 90% of α-Syn is phosphorylated at ser87 (p-ser87) and at ser129 (Anderson et al., 2006; Paleologou et al., 2010). Other sites of phosphorylation have been found on tyr residues at position 39, 125, 133, and 136.

Phosphorylation is the chemical addition of a phosphoryl group (PO^3−^) to an organic molecule. Phosphorylation and dephosphorylation (the removal of a phosphoryl group) are carried out by enzymes (e.g., kinases, phosphatases) and the processes orchestrate a plethora of cellular functions in response to external stimuli. *In vitro* and cell culture-based studies have identified a number of kinases, which phosphorylate α-Syn at ser129 and/or ser87, including casein kinase I (CKI; ser87 and ser129), casein kinase II (CKII; ser129; Okochi et al., 2000), G protein-coupled receptor kinases (GRKs 1, 2, 5 and 6; ser129; Pronin et al., 2000), leucine-rich repeat kinase 2 (LRRK2; ser129; Qing et al., 2009b), polo-like kinase (PLK; ser129; Inglis et al., 2009, Mbefo et al., 2010) protein kinase C-related kinase (PKR; ser129; Reimer et al., 2018) and LK6/Mnk2a (ser129; Zhang et al., 2015).

α-Syn phosphorylation at tyr125 can be mediated by the proto-oncogene tyrosine-protein kinase Fyn (Nakamura et al., 2001) and SRC proto-oncogene non-receptor (Src) tyr kinases such as spleen associated tyrosine kinase (Syk), the non-receptor tyrosine-protein kinase Lyn, the protein tyrosine kinase expressed by the protooncogene c-fgr (Ellis et al., 2001; Negro et al., 2002). Syk also phosphorylates α-Syn at try133 and tyr136.

Although the contribution of α-Syn pathology to LRRK2-associated PD is debated (Schneider and Alcalay, 2017) and the relevance of LRRK2-mediated α-Syn phosphorylation in PD is still to be determined, several studies reported that LRRK2 co-localizes with α-Syn in the lower brainstem of PD and LBD patients at early stages (Alegre-Abarrategui et al., 2008; Qing et al., 2009b; Zimprich et al., 2011). Still, *in vitro* studies hint that G2019S-mutant LRRK2 exhibit an improved ability to phosphorylate α-Syn on ser129 when compared to wt LRRK2 (Qing et al., 2009a).

On the other hand, the phosphatases involved in the dephosphorylation are phosphoprotein phosphatase 2A and 2C (PP2A and PP2C).

Increased ser129 α-Syn phosphorylation has been detected in PD, LBD and MSA (Kahle et al., 2000; Okochi et al., 2000; Fujiwara et al., 2002; Takahashi et al., 2003; Anderson et al., 2006). A recent study analyzing post-mortem tissue from PD and MSA patients at different disease stages reported that ser129 α-Syn phosphorylation is the dominant and earliest PTMs, while lower amounts of p-ser87 α-Syn appeared later along PD progression (Sonustun et al., 2022).

Almost all phosphorylation sites cluster at the C-terminal region of α-Syn (residues 120–140), which is involved in protein–protein, protein-ligand and protein-metal interactions, suggesting a possible role of the modification in the regulation of these functions. Only ser87 lies in the hydrophobic NAC region of α-Syn, which is essential for α-Syn aggregation and fibrillogenesis (El-Agnaf et al., 1998b).

Ser129 is the most studied phosphorylation site because it was linked with increased cytotoxicity and neuronal death (Zhang et al., 2015; Karampetsou et al., 2017; Zhong et al., 2017; Reimer et al., 2018). Furthermore, it has been described that p-ser129 enhances intracellular aggregate formation in SH-SY5Y cells (Smith et al., 2005b) and mediates cell death through activation of the unfolded protein response (UPR) pathway (Sugeno et al., 2008). Still, Karampetsou et al. (2017) observed that mice who received intrastriatal injection of p-ser129 α-Syn exhibited enhanced α-Syn pathology deposition and neurodegeneration in the substantia nigra (SN) compared to the mice injected with wild type (wt) α-Syn.

However, other studies in cellular and animal models claimed that phosphorylated α-Syn exherts a neuroprotective role (Gorbatyuk et al., 2008; Oueslati et al., 2012; Tenreiro et al., 2014; Ghanem et al., 2022). In particular, it has been demonstrated that p-ser129 phosphorylation occurs secondarily to α-Syn accumulation, reducing cytotoxicity and aggregation propensity of α-Syn (Ghanem et al., 2022). Interestingly, p-tyr125 α-Syn can also prevent α-Syn neurotoxicity and aggregation and is pivotal for ser129 phosphorylation (Kosten et al., 2014).

The role of p-ser87 is also controversial as this PTM falls in the NAC region of α-Syn, which is crucial for α-Syn aggregation and fibrillogenesis *in vitro* (Ueda et al., 1993; El-Agnaf et al., 1998a,b; Giasson et al., 2001). In addition, though p-ser87 phosphorylation is increased in the membrane fractions of *post mortem* brains of patients affected by LBD, MSA and AD and healthy controls and of rats overexpressing wt α-Syn, p-ser87 was found to reduce α-Syn membrane binding (Paleologou et al., 2010), supporting that this phosphorylation may be crucial for modulating the physiological effect of α-Syn on synaptic vesicle mobility. Moreover, the unilateral p-ser87 α-Syn overexpression in the nigrostriatal system of rats results in reduced formation of aggregates and does not exert toxicity for nigral dopaminergic neurons in contrast to what has been observed following wt α-Syn overexpression (Decressac et al., 2012; Lundblad et al., 2012; Oueslati et al., 2012; Faustini et al., 2018).

Differently, p-tyr125 was reported to decrease with aging and in PD brains, in *Drosophila melanogaster* and mice (Chen et al., 2009). As this phosphorylation has been found to reduce α-Syn oligomerization, it has been hypothesized that it may play a protective role against aggregate formation (Chen et al., 2009). On this line, Negro et al. (2002) showed that the kinase Syk phosphorylates the C-terminal tyr125 of α-Syn to block α-Syn fibrillation. Moreover, p-tyr125 facilitates the deposition of p-ser129 under physiological conditions (Kosten et al., 2014).

PLK2 has been found to phosphorylate α-Syn, but not β-or γ-syn, at ser129 in HEK293T cells and in primary neurons (Arawaka et al., 2006; Inglis et al., 2009; Mbefo et al., 2010). In particular, PLKs can phosphorylate both monomeric or fibrillary α-Syn (Waxman and Giasson, 2011) and overexpression of PLK2 enhances α-Syn turnover via the autophagic degradation pathway, thus suppressing its toxicity *in vivo* (Oueslati et al., 2013). Despite the role of PLK2 in centriole duplication and cell cycle regulation, PLK2 inhibitors do not appear to cause cytotoxicity nor genotoxicity *in vitro* or *in vivo* at doses and exposures that engage the target in rat (Fitzgerald et al., 2013), but clinical trials on PLK2 inhibitors have shown difficulties in targeting specifically PLK2 in order to avoid off-target-related side effects (Vancraenenbroeck et al., 2011).

c-Abelson tyrosine kinase (c-Abl) is a 120 kDa protein majorly known in relation to human leukemias. c-Abl is distributed in the nucleus and cytosol and is involved in a wide range of functions, including apoptosis and development of the CNS in which it affects neurogenesis, neurite outgrowth, and neuronal plasticity. Moreover, it is involved in several neurodegenerative diseases including PD (Tremblay et al., 2010; Imam et al., 2011). For instance, c-Abl is elevated in postmortem nigrostriatal region of PD patients (Ko et al., 2010; Imam et al., 2011) where it is majorly phosphorylated at tyr412 (Mehdi et al., 2016). c-Abl was found to phosphorylate parkin thus impairing its E3 ligase activity and leading to the loss of dopaminergic neurons in the SN (Ko et al., 2010). It has been described that c-Abl aberrant activation induced a progressive accumulation of α-Syn in the human A53T mutant α-Syn tg mouse model of genetic PD (Brahmachari et al., 2016) through the phosphorylation at tyr39 (Mahul-Mellier et al., 2014; Brahmachari et al., 2016; Dikiy et al., 2016), thus contributing to neurodegeneration. Furthermore, c-Abl is activated by OS (Brasher and Van Etten, 2000; Sun et al., 2000; Gonfloni et al., 2012), and in turn it disrupts antioxidant defense mechanisms driving oxidative injury (Li et al., 2004). It may thus be inferred that c-Abl inhibitors may impact on α-Syn pathology by affecting the phosphorylation and nitration state of the protein.

Consistently, Hebron et al. (2013) showed that c-Abl activation promotes α-Syn accumulation and that the treatment with nilotinib, a brain-permeable second-generation c-Abl inhibitor, developed from the first generation anticancer agent, named imatinib, favored the clearance of α-Syn, improved motor performances (Hebron et al., 2013), restored the levels of dopamine transporter (DAT) and dopamine production in the striatum as well as the expression of tyrosine hydroxylase (TH) in the SN (Hebron et al., 2013, Karuppagounder et al., 2014; Table 2).

**Table 2:** Kinase-inhibitors tested in preclinical models of PD and in clinical trials.

Target	Mechanism of action	Molecule name	Results from studies in preclinical models	Results from clinical trials	Ongoing clinical trials	Direct or indirect effect on α-Syn Phosphorylation
c-Abl	Inhibition	Nilotininb	Hebron et al. (2013), Karuppagounder et al. (2014)	Pagan et al. (2016, 2019, 2020), Simuni et al. (2021)		Direct
	Inhibition	IkT-148,009	Karuppagounder et al. (2023)		NCT04350177; NCT05424276	Direct
	Inhibition	Vodobatinib (K0706 or SCC-138)	Mandhane et al. (2019)		NCT03316820; NCT03655236	Direct

Of note, results from nilotinib clinical trials showed that the drug could reduce oligomeric α-Syn (only at 150 mg dose) as well as phosphorylated tau. Nilotinib treatment also improved dopamine metabolism in patients with PD. In particular, it increased the levels of homovanillic acid (HVA) and 3,4-Dihydroxyphenylacetic acid (DOPAC) in the CSF (Pagan et al., 2016, 2020) but without improving motor and nonmotor outcomes.

Simuni et al. (2021) run a double-blind, placebo-controlled trial on 173 PD patients. The results about safety, tolerability, adverse effects and lack of the symptomatic effect of nilotinib were in line with the study by Pagan et al. (2020). However, they could not observe changes in biomarkers. Although these evidences support that nilotinib is not suitable for further testing the collected data did not exclude the importance of c-Abl modulation in PD therapeutic strategy (Simuni et al., 2021).

The fact that no clinically meaningful benefit in PD patients in two double-blind studies was reported, is discouraging, but this can find an explanation by the fact that nilotinib does not accumulate in the brain at concentrations sufficient to inhibit c-Abl. As a competitive inhibitor of c-Abl with an IC50 of ≈48 nM it would require a sustained concentration of 150 nM to exert the adequate functions (Pagan et al., 2019). Other c-Abl inhibitors such as IkT-148,009 and vodobatinib (Table 2), are currently under development. The chronic oral treatment with IkT-148,009 was found to significantly reduce p-tyr39 and p-ser129 α-Syn levels thus preventing neurodegeneration in the brain of human A53T mutated α-Syn transgenic (tg) mice and of mice who received striatal injections of mouse recombinant α-Syn pre-formed fibrils (PFF; Karuppagounder et al., 2023). IkT-148009 is a derivative of the commercial anticancer imatinib and it has an IC50 of 33 nM for c-Abl, an improvement in potency of more than 20-fold over imatinib (Werner and Olanow, 2022). The randomized phase I/Ib study in older adult or elderly healthy volunteer was then extended to PD patients to identify the safety, tolerability, maximum tolerated dose and the pharmacokinetic profile of the molecule in single doses up to 325 mg and multiple doses up to 100 mg (Clinical trial identifier: NCT04350177). A randomized, double-blind study in non-treated PD patients is also ongoing (Clinical trial identifier: NCT05424276).

Vodobatinib, also known as K0706 or as SCC-138 is a chemical mixture of other two commercial anticancer agents (Dasatinib and Ponatinib) and it has a reported IC50 for wt c-Abl of 0.9 nM (Antelope et al., 2019). In preclinical models of PD it has been shown that it inhibited preferentially, with a sub-nanomolar potency, the protein kinase activity of c-Abl. Moreover, it increased autophagic flux, it had appreciable BBB penetration *in vivo* and protected both 1-methyl-4-phenyl-1,2,3,6-tetrahydropyridine (MPTP) mice and rats overexpressing α-Syn from nigrostriatal neuron loss (Mandhane et al., 2019).

A phase I clinical trial showed that vodobatinib was well-tolerated and allowed the selection of two doses that are likely to produce therapeutic effects (Clinical trial identifier: NCT03316820). A new double-blind, placebo-controlled phase II study is now recruiting for evaluating the safety and effectiveness of the two selected K0706 doses in people with early PD who are not receiving dopaminergic therapy (Clinical trial identifier: NCT03655236). The primary endpoints focus on changes from baseline in the sum of Movement Disorder Society-Sponsored Revision of the Unified Parkinson’s Disease Rating Scale (MDS-UPDRS) Parts 2 and 3, but among other outcome measures there will be the evaluation of CSF and blood K0706 levels and dopamine transporter single-photon emission computed tomography (DAT-SPECT).

Collectively, the above summarized studies on c-Abl inhibitors support that the use of protein kinase modulators in PD may be beneficial. Nevertheless, we need to achieve a deeper understanding of the role of the α-Syn phosphorylation and, more generally, on protein kinase and phosphatases activity in synucleinopathies, before to conclude that strategies modulating this PTM may constitute a possible therapeutic approach for this class of neurodegenerative disorders.

## 5. Nitration

Post-mortem PD brains are rich in lipid peroxidation products such as 4-hydroxyl-2-nonenal (HNE) as well as DNA and RNA oxidation products (Alam et al., 1997; Floor and Wetzel, 1998; Zhang et al., 1999). Moreover, several lines of evidence support that OS is involved in the degeneration of dopaminergic neurons in PD (Jenner and Olanow, 2006; Schapira and Tolosa, 2010).

OS is the result of a disequilibrium between the production of reactive oxygen species (ROS) or reactive nitrogen species (RNS) and the system for the detoxification leading to the production of free radicals byproducts that damage proteins, lipids, nucleic acids and organelles (Ryan et al., 2014). Although the brain represents only 2% of the body weight, it consumes 20% of the total body oxygen (Quastel and Wheatley, 1932; Magistretti and Pellerin, 1996), which is majorly converted in ROS. To defend against oxidative injuries, cells own a series of enzyme-based antioxidant mechanisms, such as glutathione (GSH), SOD and DJ-1. However, these systems are feeble in preventing the damage. In particular, nigral dopaminergic neurons are particularly sensitive to oxidative injuries as they own long, highly branched axons with a huge number of release sites that renders these cells bioenergentically demanding and at risk of developing mitochondrial OS (Pissadaki and Bolam, 2013). Nigral dopaminergic neurons also own a pacemaking activity characterized by broad and slow action potentials in the absence of synaptic input (Grace and Bunney, 1983). This activity engages continuously L-type Ca^2+^ channel, creating a basal mitochondrial OS in SN dopaminergic neurons (Guzman et al., 2010) and elevating intracellular Ca^2+^ levels (Wilson and Callaway, 2000; Chan et al., 2007). In light of the fact that cytoplasmic Ca^2+^ controls a huge number of pathways within a cell, its presence inside a neuron must be strictly controlled, and it is rapidly sequestered or pumped back in an ATP-dependent manner, thus resulting highly energy demanding (Wilson and Callaway, 2000). Still, dopamine turnover by monoamine oxidases (MAO) is involved in the production of cytotoxic free radicals, causing the death of dopaminergic neurons (Greenamyre and Hastings, 2004). Among them, the MAO-derived dopamine catabolite 3,4-dihydroxyphenylacetaldehyde (DOPAL) exhibits an enhanced reactivity towards proteins especially at synaptic terminals (Rees et al., 2009) and has been recently found to contribute to the initiation of α-Syn-dependent impaired proteostasis and degeneration of neuronal projections in different experimental models of PD (Masato et al., 2023).

Consistently, it is well established that mitochondria dysfunction is crucially involved in the pathogenesis of PD. This is also supported by the fact that several gene mutations implicated in familial and idiopathic forms of PD are located on loci encoding for mitochondria-linked proteins (Moore et al., 2005; Abou-Sleiman et al., 2006; Schapira, 2008). Moreover, postmortem studies on the SN of sporadic PD patients reported a decreased activity of mitochondrial complex I and higher levels of iron in the SN (Mann et al., 1994; Keeney et al., 2006). Free iron is toxic since it can donate or accept an electron from neighboring molecules and cause damage to cellular components and it can create ROS through the Fenton and Haber-Weiss reaction, in which ferric iron (Fe^3+^) and ferrous iron (Fe^2+^) react with superoxide and hydrogen peroxide to form hydroxyl radical (Beard and Connor, 2003; Jomova and Valko, 2011; Eid et al., 2017). Neuromelanin, the dark colored granular pigment present in the dopaminergic neurons of the SN, has the ability to chelate metals, in particular the ferric Fe^3+^ form (Gerlach et al., 2003), thus blocking the Fenton reaction and protecting the cells from hydroxyl radical production. The huge increase of iron found in SN of PD brains might saturate the iron-chelating site of neuromelanin, increasing the production of free radical species. Finally, neuroinflammation can also contribute to OS in the PD brain (Mosley et al., 2006; Picca et al., 2020; Teleanu et al., 2022).

The interplay between α-Syn and OS is still not fully elucidated. *In vitro* and *in vivo* studies support that increased OS in the brain may promote α-Syn aggregation (Paxinou et al., 2001), but α-Syn itself can increase ROS production (Junn and Mouradian, 2002; Winklhofer and Haass, 2010) or it can bind to mitochondrial complex I causing mitochondrial dysfunction in turn favoring OS (Chinta et al., 2010; Winklhofer and Haass, 2010; Wilkaniec et al., 2013).

Nitrated α-Syn can be easily formed under OS conditions. α-Syn has four tyr residues, placed in positions 39 (at the N-terminal region), 125, 133, and 136 (at the C-terminal region). The positions of the nitration sites suggest a possible modulation of membrane binding ability (Hodara et al., 2004) and protein–protein and protein-metal interactions. α-Syn is sensitive to the presence of nitrating agents and the presence of peroxynitrite not only induces the deposition of 3-nitrotyrosines but also the formation of 3,3-dityrosine via the oxidation of tyr residues, which results in α-Syn dimers and oligomer formation (Souza et al., 2000). Danielson et al. (2009) demonstrated a selective 9-fold increase in nitration on tyr39 of α-Syn in oxidative cellular model of PD. In addition, nitration of tyr39 induces high rate of oligomerization (Hodara et al., 2004) similarly to n-tyr125 that contributes to α-Syn dimer formation upon the exposure of recombinant α-Syn to nitrating agents (Takahashi et al., 2002).

Interestingly, a recent study analyzing post-mortem tissue from PD and MSA patients at different disease stages reported that ser129 α-Syn phosphorylation is the dominant and earliest PTMs, followed by tyr39 nitration, while lower amounts of p-ser87 α-Syn appeared later along PD progression (Sonustun et al., 2022). However, in the MSA brain glial cytoplasmatic inclusions, neuronal inclusions and small threads are mainly positive for tyr39 nitrated while ser129 α-Syn can be mainly detected in Schwan cell and neuronal inclusions (Sonustun et al., 2022; Wakabayashi et al., 2022).

Nitrated α-Syn monomers and dimers have been shown to accelerate fibril formation while nitrated α-Syn oligomers inhibit this process (Hodara et al., 2004). This supports that improving the amount of nitrated α-Syn oligomers may delay the formation of mature fibrils. This notwithstanding, as we still ignore whether fibrils or oligomers are the major neurotoxic species in PD, it is hard to predict whether this may be beneficial or detrimental.

Nevertheless, it may be feasible that antioxidant supplementation may be used to reduce α-Syn nitration. In this framework, some antioxidant schemes have been attempted, such as the supplementation of vitamin C, E and β-carotene as well as an adequate diet (Percario et al., 2020). Vitamin A and its precursor β-carotene, have been involved in the destabilization of fibrillary α-Syn *in vitro* (Ono et al., 2004; Ono and Yamada, 2007). Vitamin E (i.e., α-tocopherol) and Vitamin C (i.e., ascorbic acid) are antioxidants that are thought to have a protective effect by either reducing or preventing oxidative damage, preventing or interacting directly with free radicals, respectively. A lot of studies tried to investigate the relation between the intake of vitamins and the protection from PD, but they generated only conflicting results (Kieburtz et al., 1994; Hellenbrand et al., 1996; Morens et al., 1996; de Rijk et al., 1997; Scheider et al., 1997; Etminan et al., 2005; Miyake et al., 2011; Hughes et al., 2016; Schirinzi et al., 2019; Zhao et al., 2019). It has been demonstrated that NXP031, a new compound composed of aptamin C and vitamin C, blocks α-Syn aggregation in the hippocampus of AAV-human α-Syn-injected mice (Song et al., 2022). Similarly, also vitamin B12 was found to inhibit α-Syn fibrillogenesis in *in vitro* models (Jia et al., 2019).

Recent studies on MPTP *in vivo* and *in vitro* models support that γ-and δ-tocotrienol reduces dopaminergic neuron toxicity and improves motor performances through estrogen receptor/PI3K/Akt signaling pathway activation, hence in an antioxidant-independent way (Matsura, 2019). The supplementation of α-and δ-tocotrienol significantly ameliorates motor behavior and prevents the loss of nigra dopaminergic neurons and striatal fibers and neuroinflammation in 6-Hydroxydopamine (6-OHDA)-injected rats (Kumari et al., 2021). The vitamin E family compound tocotrienol is currently under study as a potential agent to delay motor symptoms in PD patients at Hoehn & Yahr stage 2 in a phase II clinical trial (Clinical trial identifier: NCT04491383; Table 3).

**Table 3:** List of OS modulators tested in preclinical models of PD and in clinical trials.

Target	Mechanism of action	Molecule name	Results from studies in preclinical models	Results from clinical trials	Ongoing clinical trials	Direct or indirect effect on α-Syn Nitration
Vitamin E	Supplementation	Tocotrienol	Kumari et al. (2021)		NCT04491383	N/A
Iron	Removing	Deferiprone		Devos et al. (2022)		Indirect (reduces a-Syn aggregates)
GSH	Supplementation	N-acetylcisteine	Monti et al. (2016)	Holmay et al. (2013), Monti et al. (2016)		Indirect (reduces a-Syn aggregates)
CoQ10	Supplementation	Coenzime Q10	Beal (1998), Horvath et al. (2003), Menke et al. (2003), Sherer et al. (2003), Gille et al. (2004), Shavali et al. (2004)	Shults and Schapira (2001), Parkinson Study Group et al. (2014)		Indirect
		Idebenone	Avci et al. (2021), He et al. (2021)		NCT03727295; NCT04152655	Indirect (induces a-Syn degradation)
Nrf2	Supplementation	Sulforaphane	Uddin et al. (2020)		NCT05084365	Indirect
Several pathways involved in oxidative stress	Inhibits pro-oxidative mechanism and enhances anti-oxidative systems	Melatonin	Li and Pelletier (1995), Gilad et al. (1998), Crespo et al. (1999), Reiter et al. (2002a,b, 2003, 2005), Dong et al. (2003), Lopez-Burillo et al. (2003), Rodriguez et al. (2004, 2007), Deng et al. (2006), Lopez et al. (2006), Winiarska et al. (2006), Sudnikovich et al. (2007)	Kunz and Bes (1999), Boeve et al. (2001), Kunz et al. (2004)	NCT02768077; NCT03258294; NCT02789592; NCT02359448; NCT04287543	Indirect

This notwithstanding, a multicenter, phase II, randomized, double-blind trial in early drug-naïve PD patients evaluating the efficacy of the iron chelator deferiprone (Table 3) on disease progression indicate that 36 weeks of therapy with deferiprone could remove specifically, safely and gradually the iron content in the nigrostriatal system of PD patients but it worsened the progression of symptoms (Devos et al., 2022). Studies on the efficacy of deferiprone in experimental *in vivo* models of synucleinopathies led to conflicting results. Indeed, human A57T α-Syn tg mice showed improvement in behavioral performances upon deferiprone treatment but without reduction of α-Syn aggregation (Carboni et al., 2017), while deferiprone treated mouse model of MSA exhibited rescued motor performance, higher neuronal survival and reduced density of α-Syn aggregates in SN (Shukla et al., 2021).

Another possible strategy to counteract OS is based on GSH rebalancing. In particular, since GSH is neither able to pass the blood brain barrier (BBB) nor the cellular membrane of neurons, the dietary supplementation of this enzyme is not possible. However, cysteine, which is rate-limiting in the GSH synthesis pathway, crosses both the BBB and most cell membranes. Therefore, cysteine and its derivative N-acetylcysteine have been investigated as a possible dietary supplementation to implement GSH amount, with several clinical trials ongoing (Table 3). Intravenous N-acetylcysteine injection increased blood GSH redox ratios in PD and healthy subjects and magnetic resonance spectroscopy (MRS) showed higher brain GSH concentrations in all subjects. This supports that it is possible to directly monitor GSH levels that could help during clinical trial to determine the activities and the doses of this antioxidant therapy (Holmay et al., 2013).

Another study aimed at assessing the effect of N-acetylcysteine on human embryonic stem cells-derived midbrain dopaminergic neurons treated with rotenone and on PD patients and showed that N-acetylcysteine exposure significantly improved the survival of midbrain dopaminergic neurons treated with rotenone (Monti et al., 2016). Furthermore, Dopamine Transporter scan (DaTscan) analysis on patients treated for 3 months with N-acetylcysteine resulted in increased DAT binding in the caudate and putamen (Monti et al., 2016). These results support a potential direct effect of N-acetylcysteine (Table 3) on the dopamine system in PD patients, but we still ignore whether this compound affects α-Syn nitration state though N-acetylcysteine has shown protective effects against the damage in dopaminergic terminals concomitant with a reduction in α-Syn levels in transgenic mice (Clark et al., 2010).

Coenzime Q10 (CoQ10) is a key component of the electron transport chain that leads to decreased free radical generation, and, in its reduced form, acts as a powerful antioxidant (Shults, 2005). CoQ10 levels were altered in PD cases (Matsubara, 1991; Shults et al., 1997; Molina et al., 2002) with a significant increase in the percentage of oxidized CoQ10 in affected patients (Sohmiya et al., 2004). Numerous studies in *in vitro* and *in vivo* models of PD demonstrated that CoQ10 protects neurons against MPTP and rotenone toxicity (Beal, 1998; Horvath et al., 2003; Menke et al., 2003; Sherer et al., 2003; Gille et al., 2004), and 1-Benzyl-1,2,3,4-tetrahydroisoquinol (Shavali et al., 2004; Table 3).

A randomized, double-blind, placebo-controlled, multicenter phase II study in early PD examined the effects of 300, 600, and 1,200 mg per day of CoQ10 vs. placebo. CoQ10 supplementation decreased functional decline in participants and increased platelet mitochondrial complex I and II/III activities. These results suggested a possible disease-modifying effect (Shults and Schapira, 2001). Based on these results, in a phase III study, the group tested whether high doses (1,200 and 2,400 mg/d) of CoQ10 could slow functional decline in early PD. The results showed that CoQ10 could be safely administered to patients with early PD, however no therapeutic efficacy was demonstrated (Parkinson Study Group et al., 2014).

The hydrophilic analogue of CoQ10, idebenone, is well-known antioxidant compound with better pharmacological properties. Clinical safety of idebenone was well described, and the molecule is currently used to treat Freidrich’s ataxia and AD (Orsucci et al., 2011; Montenegro et al., 2018). Two clinical trials assessing the efficacy and safety of idebenone in PD are currently ongoing (Clinical trial identifier: NCT03727295; NCT04152655) and results obtained on PD models are encouraging (Table 3). Indeed, idebenone improved motor coordination and locomotor activity while decreasing TH-positive neurons damage, lipid peroxidation, ferroptosis and other OS markers in rotenone-induced PD models (Avci et al., 2021). Moreover, idebenone activated autophagy and promoted α-Syn degradation by suppressing the AKT/mTOR pathway in SH-SY5Y overexpressing the A53T mutant form of α-Syn (He et al., 2021). This mechanism appears unusual for this compound, but recently idebenone has been demonstrated to act as cytoprotective molecule activating fundamental pathways rather than by functioning as a direct antioxidant agent (Gueven et al., 2021; He et al., 2021).

A new interesting agent for OS modulation is sulforaphane, a phytocompound belonging to the isothiocyanate family and owning lipophilic nature and a molecular size that makes it highly bioavailable (Schepici et al., 2020; Uddin et al., 2020). Its molecular target is nuclear factor erythroid 2 related factor 2 (Nrf2), which is a crucial controller of enzymes involved in antioxidation and detoxification of xenobiotics (Eggler et al., 2008; Zhang et al., 2013; Stefanson and Bakovic, 2014; Sajja et al., 2017). *In vitro* studies on cellular models of PD treated with sulforaphane showed reduced OS, cell damage and death (Uddin et al., 2020; Table 3). In line with the *in vitro* studies, *in vivo* experiments demonstrated that in C57BL/6 mice sulforaphane administration improved motor deficits and counteracted nigrostriatal dopaminergic neurons degeneration and apoptosis attenuating OS and neuroinflammation (Uddin et al., 2020). A phase II clinical trial is currently ongoing to evaluate the efficacy and safety of sulforaphane in PD patients (Clinical trial identifier: NCT05084365).

An interesting molecule to counteract OS is melatonin, a hormone produced endogenously by pineal gland and other tissues. It regulates circadian cycle and also plays a relevant role in neuroprotection, anti-inflammation and anti-oxidation. For all these reasons, it has been considered as a candidate for PD therapy (Table 3). Melatonin is an indoleamine and it can yield electron easily, hence it is a potent reducer agent. It acts as a scavenger for oxygen-and nitrogen-based reactive molecules (Reiter et al., 2002a,b, 2003; Lopez-Burillo et al., 2003; Sudnikovich et al., 2007) and it works as an inhibitor of inducible NO synthase (iNOS; Gilad et al., 1998; Crespo et al., 1999; Dong et al., 2003; Rodriguez et al., 2004, 2007; Lopez et al., 2006). The ability to interact with iNOS and peroxinitrite is the one that makes melatonin a special candidate for the treatment of OS as none of the previous mentioned antioxidant is able to exert this action. It has been demonstrated that melatonin also helps antioxidant enzymes, including SOD and GPx, stimulating the production of GSH (Rodriguez et al., 2004; Reiter et al., 2005; Winiarska et al., 2006). In addition, melatonin has been found to inhibit cyclooxygenase-2 reducing the severity of inflammation (Deng et al., 2006). In particular, it ameliorates inflammation blocking tumor necrosis factor-α (TNF-α; Li and Pelletier, 1995; Reiter et al., 2003) and it impacts on mitochondrial respiration, protecting both proteins of electron transport chain and mitochondrial DNA from oxidative damage (Reiter et al., 2008). Interestingly, melatonin has been found to reduce α-Syn secretion in rat adipose-derived mesenchymal stem cells (Ibrahim et al., 2022). Several phase II and III clinical trials are evaluating the effect of melatonin on sleep disturbances in PD patients (Clinical trial identifiers: NCT02768077; NCT03258294; NCT02789592; NCT02359448; NCT04287543; Table 3). Interestingly, trial NCT04287543 aimed at following the activity of mitochondrial complex I, the levels of MDA and 4-hydroxyalkene and the production of NO among the secondary outcome measures, but it was withdrawn because of COVID-19 pandemic. Other studies on exogenous melatonin investigated the effect of the molecule on rapid eye movement (REM) sleep behavior disorder (RBD), which is a prodromal sign for PD. Among them, the study by Kunz et al. (2004) demonstrated that medical melatonin increased REM sleep percentage to normal levels in patients with reduced REM sleep duration and re-organized REM sleep episode length during night-time sleep. The effect lasted for several weeks after the discontinuation of the therapy. Other studies reported a resolution of clinical RBD symptoms lasting for up to 3 years after discontinuation of melatonin treatment (Kunz and Bes, 1999; Boeve et al., 2001; Kunz et al., 2004).

It is worth considering that unfortunately the limitations offered by OS targeting therapeutic strategies are challenging. Moreover, despite OS is common to several diseases, it rarely constitutes the primary cause of a disease, supporting that the use of an antioxidant may have mild impact on pathology progression. Moreover, *in vitro* and *in vivo* evidences demonstrated that endogenous antioxidants support the progression of different types of tumors (Singh et al., 2008; DeNicola et al., 2011; Sayin et al., 2014; George and Abrahamse, 2020; Harris and DeNicola, 2020). This effect is even greater in older people, where the activation of Nrf2 pathway, which usually is chemopreventing, can be deleterious and it could predispose for tumor progression (Forman and Zhang, 2021). Still, all classical antioxidants, excluding melatonin, are potential electron donors and they exhibit both reduced and oxidized forms. In general, these oxidized molecules should be regenerated to the reduce form through a process of recycling that consumes GSH to be exploited or through a redox reaction that, eventually, oxidizes other molecules. This means that the classical antioxidant may act as prooxidant molecules, causing other damages. However, the toxic concentrations of most of these prooxidant regenerated compounds are extremely high and their toxic potential appears negligible.

Another issue is related to the discrepancy that exists in the ratio of *in vitro* vs. *in vivo* exogenous agents. In general, in *in vitro* studies free radicals are produced at much greater rates than what would be observed in real physiological or pathological conditions (Forman et al., 2014). In addition, antioxidant defenses may not be able to reach effective concentrations *in vivo*. Therefore, it is hard to think that antioxidant approaches may significantly impact on PD progression though we cannot exclude that they may contribute in reducing α-Syn nitration.

## 6. Acetylation

Protein acetylation is one of the major PTM found in eukaryotes, in which the acetyl group from acetyl coenzyme A is transferred to a specific site on a polypeptide chain. Acetylation is mostly known for the role on gene transcription regulation, indeed through the reversible accumulation of acetylation on the lysines (ac-lys) of the histones, the transcription is activated.

In humans, 80–90% of all proteins become co-translationally acetylated at their N-terminal (Nt) of the nascent polypeptide chains (Arnesen, 2009; Aksnes et al., 2015) in an irreversible way. Nt-acetylation is a general mechanism for stabilizing α-helical structures in both proteins and peptides (Chakrabartty et al., 1993), and makes α-Syn resistant for amyloid aggregation enhancing both protein–protein and protein-membrane interaction (Bartels et al., 2014). Indeed, recent findings indicate that all the *in vivo* detectable α-Syn is post-translationally modified by an acetyl group attached to the amino group of the first N-terminal amino acid (Anderson et al., 2006; Bartels et al., 2011; Ohrfelt et al., 2011). This modification alters the charge and structure of α-Syn molecules affecting their interaction with lipid membranes, as well as their aggregation process (Bell et al., 2022a,b, 2023). It has been found that ac-lys impacts on α-Syn aggregation (Fauvet et al., 2012; Kang et al., 2012; Gruschus et al., 2013; Bu et al., 2017; de Oliveira et al., 2017) and that acetylated α-Syn and α-tubulin inhibit oligomers formation (Kazantsev and Kolchinsky, 2008). Interestingly, studies demonstrated that increases in histone acetylation are disease-dependently associated with PD progression (Park et al., 2016; Harrison et al., 2018; Toker et al., 2021) and histone-3 or-4 hyperacetylation is a key epigenetic change in dopaminergic neurons exposed to other PD-related neurotoxins. Conversely, the deacetylation of histones operated by histone deacetylase (HDAC) is implicated in the control of α-Syn toxicity. The activity of HDAC6 has been linked with PD pathogenesis (Lemos and Stefanova, 2020) and HDAC6 is highly expressed in LB in PD patients’ brain sections, indicating that HDAC6 may play a key role in the clearance of those misfolded and aggregated protein (Kawaguchi et al., 2003; Du et al., 2010; Richter-Landsberg and Leyk, 2013). Indeed, HDAC6 decreased activity is an essential factor for impaired autophagic flux in PD pathophysiology (Wang et al., 2019). Several studies demonstrated that the inhibitors of HDAC worsen the motor abilities of mice and exacerbate cell death in primary neuron cells (Du et al., 2014), while other demonstrated that HDAC inhibitors restore axonal transport and motor behavior (Godena et al., 2014; Pinho et al., 2016), reduce ROS production, and alleviate dopaminergic neurotoxicity (Jian et al., 2017). Other studies demonstrated the protective effect of pan-HDAC inhibitors such as valproic acid, sodium butyrate, phenylbutyrate, suberoylanilide hydroxamic acid and trichostatin A in *in vitro* and *in vivo* models of PD acting through different mechanism listed in Table 4 (Gardian et al., 2004; Chen et al., 2007, 2012; Wu and Guo, 2008; Kidd and Schneider, 2010, 2011; Zhou et al., 2011, 2014; Rane et al., 2012; St Laurent et al., 2013; Harrison et al., 2015; Suo et al., 2015; Sharma et al., 2015a; Kim et al., 2019; Getachew et al., 2020; Hsu et al., 2020). The specific inhibitors of HDAC1, 2 and 3, RGFP109, K560, K-856, MS-275, MC-1568, and LMK235 also showed neuroprotection against α-Syn toxicity (Table 4; Johnston et al., 2013, Formisano et al., 2015, Choong et al., 2016, Hirata et al., 2018, Mazzocchi et al., 2021).

**Table 4:** HDAC-modulators tested in preclinical models of PD and in clinical trials.

Target	Mechanism of action	Molecule name	Results from studies in preclinical models	Results from clinical trials	Ongoing clinical trials	Direct or indirect effect on α-Syn Acetylation
HDAC	Inhibition	Valproic acid	Chen et al. (2007), Wu and Guo (2008), Kidd and Schneider, (2011), Harrison et al. (2015); Kim et al. (2019), Hsu et al. (2020)			Indirect (JNK Pathway)
		Sodium butyrate	Rane et al. (2012), St Laurent et al. (2013), Sharma et al. (2015a), Getachew et al. (2020)			Indirect (activates autophagy)
		Phenylbutyrate	Gardian et al. (2004), Zhou et al. (2011)		NCT02046434	Indirect
		Suberoylanilide hydroxamic acid	Chen et al. (2012)			Indirect
		Trichostatin A	Zhou et al. (2014), Suo et al. (2015)			Indirect
HDAC1, 2 and 3	Inhibition	RGFP109	Johnston et al. (2013)			Indirect
		K560, K-856	Choong et al. (2016), Hirata et al. (2018)			Indirect
		MS-275, MC-1568	Formisano et al. (2015)			Indirect
		LMK235	Mazzocchi et al. (2021)			Indirect
SIRT1	Activation	Resveratrol	Albani et al. (2009), Guo et al. (2016), Zhang et al. (2018), Chau et al., 2021; Arbo et al. (2020), Mehringer et al. (2022)			Indirect
	Activation	Nicotinamide riboside	Schondorf et al. (2018)	Brakedal et al. (2022)		Indirect

On this line, a recent phase I clinical trial investigated whether phenylbutyrate (Table 4) can increase the removal of α-Syn from the brain into the bloodstream (Clinical trial identifier: NCT02046434), but results are not available yet.

Sirtuins (SIRT) are nicotinamide adenine dinucleotide (NAD^+^)-dependent HDAC, proteins implied in neurodegenerative disorders (Satoh and Imai, 2014). In mammals, there are seven members of the SIRT family: SIRT1-SIRT7. SIRT2 is the most abundant SIRT in the brain and its levels increase with aging (Maxwell et al., 2011). De Oliveira et al. (2017) recently described that SIRT2 interacts with and removes acetyl groups from α-Syn. They also demonstrated both *in vitro* and *in vivo* that the inhibition of SIRT2 decreased α-Syn toxicity (Outeiro et al., 2007; de Oliveira et al., 2017).

On the other hand, SIRT1 increases lifespan in mammals (Cohen et al., 2004), promotes mitochondrial biogenesis (Wenz, 2013), protects against neurodegeneration (Kim et al., 2007) and mitigates α-Syn pathology through the induction of the chaperone heat shock protein 70, which prevents the misfolding or clear the aggregates by degradation (Donmez et al., 2012). By reducing signs of aging, the SIRT1-activating drugs, such as resveratrol may have a role in the counteract of neurodegenerative diseases (Barger et al., 2008; Pearson et al., 2008). Indeed, resveratrol and its derivatives are able to alleviate motor and cognitive deficits and neuropathology in different mouse model of PD (Table 4; Guo et al., 2016, Zhang et al., 2018) and to reduce α-Syn toxicity and OS in *in vitro* models of the pathology (Albani et al., 2009; Arbo et al., 2020; Chau et al., 2021). Interestingly, though the bioavailability and brain penetration of resveratrol are problematic, some modified forms of this molecule have been developed to overcome these issues (Intagliata et al., 2019) and it has been demonstrated that one of the more bioavailable forms of resveratrol acts as a protein aggregation suppressor *in vitro* and *in vivo* (Mehringer et al., 2022).

The upstream regulation of SIRT through a replenishment of NAD within the brain has been attempted through the nicotinamide riboside supplementation. Brakedal et al. (2022) summarized the double-blinded, randomized, placebo-controlled phase I study of nicotinamide riboside in which they demonstrated a mild improvement in motor ability and a neuroprotective effect that was previously shown in murine, *Drosophila melanogaster* and induced pluripotent stem cells-based experimental models of noise induced hearing loss, amyotrophic lateral sclerosis, depression and PD (Table 4; Brown et al., 2014, Sorrentino et al., 2017, Schondorf et al., 2018, Han et al., 2020, Harlan et al., 2020, Xie et al., 2020). Nicotinamide riboside may target multiple processes implicated in the pathophysiology of the disease by upregulating the expression of genes involved in mitochondrial respiration, oxidative damage response, lysosomal and proteasomal function as well as by downregulating inflammatory cytokines in the central nervous system (Canto et al., 2012; Gong et al., 2013; Mehmel et al., 2020; Brakedal et al., 2022). In addition, it is possible that nicotinamide riboside may mitigate epigenomic dysregulation in PD by regulating histone acetylation. Increasing neuronal NAD levels would boost the activity of the NAD-dependent histone deacetylases of the SIRT family, potentially ameliorating histone hyperacetylation in PD.

## 7. O-GlcNAcylation

O-linked N-acetylglucosamine (O-GlcNAc) is a form of protein glycosylation in which N-acetylglucosamine (GlcNAc) residues are O-linked to ser and threonine (thr) hydroxyl groups of proteins (Butkinaree et al., 2010). The enzymes which control the levels of GlcNAc are O-GlcNAc transferase (OGT) which attaches O-GlcNAc and O-GlcNAcase (OGA), which instead removes the O-GlcNAc (Bond and Hanover, 2013).

O-GlcNAcylation reduces the aggregation propensity and the toxicity of amyloidogenic proteins including and α-Syn (Marotta et al., 2015; Levine et al., 2017; Lewis et al., 2017). α-Syn has several O-GlcNAcylation sites (Cole and Hart, 2001), especially located in the NAC region of the protein (Marotta et al., 2015; Levine et al., 2017, 2019; Lewis et al., 2017). The O-GlcNAcylation at thr72 of α-Syn decreases aggregation propensity and toxicity in cultured cells (Marotta et al., 2015). Moreover, O-GlcNAcylation hampers the cleavage of α-Syn by calpain (Levine et al., 2017), a process involved in the formation of aggregates, and is implicated in the modulation of endocytic and autophagic pathways (Dufty et al., 2007). In addition, it has been demonstrated that pharmacological inhibition or the knockdown of OGA hampers α-Syn pre-formed fibrils internalization (Tavassoly et al., 2021).

Selective inhibitors of OGA are of interest for their potential to reduce the aggregation of the amyloidogenic proteins within brain (Selnick et al., 2019). In this context, thiamet G, a brain permeable molecule, has been shown to increase cerebral O-GlcNAc levels to hamper neurodegeneration and reduce phosphorylation and aggregation of tau (Liu et al., 2004a; Yuzwa et al., 2008; Gong et al., 2012). Moreover, thiamet G improves behavioral features in preclinical models of tauopathies (Yuzwa et al., 2008, 2012, 2014a,b; Yu et al., 2012; Borghgraef et al., 2013; Graham et al., 2014; Hastings et al., 2017). A novel, highly potent and selective OGA inhibitor, MK-8719, has been developed and showing promising results in *in vitro* and *in vivo* tauopathies model. The OGA inhibitor ASN120290, that has been recently assigned the Orphan Drug Designation for the treatment of progressive supranuclear palsy (PSP) by the Food and Drug Administration has granted to ASN120290 reduced neurofibrillary tangles in mouse model of tauopathy. Permanne et al. (2022) demonstrated that the administration of ASN120290 enhance α-Syn O-GlcNAcylation and slows the progression of motor impairment in a α-Syn tg mouse model of PD (Table 5). In June 2021, a phase I first-in-human trial assessing the diffusion of ASN121151 to the CNS and the safety and pharmacokinetic profile in elderly healthy and AD subjects has been started (Clinical trial identifier: NCT04759365). Furthermore, a multiple ascending doses PET study is currently ongoing to investigate the brain occupancy of OGA and the pharmacodynamic response in peripheral blood mononuclear cells after repeated doses of ASN121151 to healthy subjects (Clinical trial identifier: NCT05725005; Table 5).

**Table 5:** Inhibitors of OGA tested in preclinical models of PD or in clinical trials.

Target	Mechanism of action	Molecule name	Results from studies in preclinical models	Results from clinical trials	Ongoing clinical trials	Direct or indirect effect on α-Syn O-GlcNAcylation
O-GlcNAcase	Inhibition	ASN120290	Permanne et al. (2022)			Direct
		ASN121151			NCT04759365; NCT05725005	Direct

## 8. Glycation

In the context of sugar-based modifications we can find glycation. Glycation is a non-enzymatic reaction that proceeds under hyperglycemia and during aging. Through the Maillard reaction the reduced carbohydrates and amino compounds form the intermediate Amadori products which in turn break down, thus creating a variety of different carbonyl and dicarbonyl intermediate products, including glyoxal and methylglyoxal (MGO) that are able to bound to the proteins (Hodge, 1955). Lastly, higher molecular weight species or advanced glycation end products (AGEs) can be formed from these lower molecular weight species (Henning and Glomb, 2016). These reactions are generally rather slow and their end products are very stable (Henning and Glomb, 2016). Therefore, short lived proteins are usually not involved in this process, however long-lived proteins, such as α-Syn can be modified in AGEs (Ahmed, 2005; Vicente Miranda and Outeiro, 2010). AGEs colocalize with α-Syn in LB in the SN (Munch et al., 2000) and glycated α-Syn has been identified in brain tissue from PD patients (Vicente Miranda et al., 2017b). MGO reacts with α-Syn to form oligomers, increasing the toxicity (Vicente Miranda et al., 2017b). In addition, diabetes is associated with the accumulation of AGEs (Kopytek et al., 2020) and patients with type 2 diabetes mellitus experience an increased risk to develop PD (Yang et al., 2017; Vaccari et al., 2021), indicating a possible insulin-modulating role in this latter condition. Both diabetes and PD are characterized by altered homeostasis of sugar metabolism (Dunn et al., 2014; Shamsaldeen et al., 2016; Trezzi et al., 2017). Interestingly, antidiabetic drugs have been suggested to exert a neuroprotective role both in PD models and in patients (Konig et al., 2018; Iravanpour et al., 2021). For instance, insulin modulates α-Syn expression and aggregation (Sharma et al., 2015b,c), regulates vesicular monoamine transporter 2 (VMAT2; Kong et al., 2020) and intranasal administration of insulin ameliorated mitochondrial function, motor impairment and dopaminergic neuron death in a rat model of PD (Iravanpour et al., 2021).

Glucagon-like peptide-1 (GLP1) is secreted in response to ingestion and absorption, preferably of carbohydrates and fats (Drucker and Nauck, 2006; Wu et al., 2015; Nauck and Meier, 2018). The binding of GLP1 to its receptor (GLP1R) induces the glucose-dependent pancreatic insulin secretion (Flock et al., 2007; Holst, 2007). It has been demonstrated that agonists (GLP1RA) such as exendin-4 (Ex-4) can regulate several functions related to neurodegeneration, OS and neurogenesis (Kim et al., 2017). Consistently, Ex-4 and derivatives showed beneficial effects in PD animal models (Bertilsson et al., 2008, Rampersaud et al., 2012, Liu et al., 2015, Palleria et al., 2017, Chen et al., 2018, Elbassuoni and Ahmed, 2019, Zhang et al., 2021; Table 6). Indeed, it has been demonstrated that GLP1RA ameliorates MPTP-induced neurotoxicity acting on mitophagy flux, OS and α-Syn aggregation in both the MPTP-mouse model of PD (Lin et al., 2021) and in α-Syn transgenic mice (Yun et al., 2018).

**Table 6:** Glycation-modifying agents tested in preclinical models of PD and in clinical trials.

Target	Mechanism of action	Molecule name	Results from studies in preclinical models	Results from clinical trials	Ongoing clinical trials	Direct or indirect effect on α-Syn Glycation
Glucagon-like peptide-1 receptor	Activation	Exendin-4	Bertilsson et al. (2008), Rampersaud et al. (2012), Liu et al. (2015), Palleria et al. (2017), Chen et al. (2018), Elbassuoni and Ahmed (2019), Zhang et al. (2021)			Indirect
	Activation	Exenatide		Aviles-Olmos et al. (2013, 2014), Athauda et al. (2017, 2018, 2019a,b)		Indirect
	Activation	Liraglutide			NCT02953665	Indirect
	Activation	Semaglutide			NCT03659682	Indirect
	Activation	Lixisenatide			NCT03439943	Indirect
DDP4	Inhibition	Vildaglitpin, Saxagliptin, Linaglitptin and Sitaglitpin	Abdelsalam and Safar (2015), Nassar et al. (2015), Kabel et al. (2018)	Svenningsson et al. (2016), Brauer et al. (2020), Jeong et al. (2021)		Indirect
Glucagon	Inhibition	Metformin	Katila et al. (2017), Parekh et al. (2022), Ryu et al. (2020)	Wahlqvist et al. (2012), Ping et al. (2020)		Indirect
		Mitoglitazone		Brauer et al. (2015, 2020), Brakedal et al. (2017)		Indirect
Advanced glycation end products levels	Reducing	Thiamine		Costantini et al. (2013, 2015), Karachalias et al. (2010)		Indirect
Methylglyoxal	Scavenging	Tenilsetam and aminoguanidine	Vicente Miranda et al. (2017a,b)			Indirect
Advanced glycation end products levels	Reducing	Telmisartan	Sato et al. (2014)			Indirect

Phase II clinical trials assessing the effect of 12 or 24 months treatments with exenatide, a synthetic Ex-4 derivative, showed cognitive and motor benefits which persisted for 12 months after drug washout in moderate PD patients (Aviles-Olmos et al., 2013, 2014; Table 6). In a next randomized, placebo-controlled, double-blind trial the authors analyzed the improvements of exenatide treated PD patients regarding motor abilities (Athauda et al., 2017), mood and cognition (Athauda et al., 2018). A *post hoc* analysis showed that younger patients with lower MDS-UPDRS-2 scores and tremor-dominant phenotype had the best response to exenatide (Athauda et al., 2019b). Moreover, there was a positive trend in obese patients or those with insulin resistance (Athauda et al., 2019a). Several other trials are evaluating other GLP1RA such as liraglutide, semaglutide or lixisenatide (Clinical trial identifier: NCT02953665; NCT03659682; NCT03439943; Table 6).

Dipeptidyl peptidase 4 (DDP4) inhibitors such as Vildaglitpin, Saxagliptin, Linaglitptin and Sitaglitpin have also been tested in animals as blockers of peripheral GLP1 degradation (Abdelsalam and Safar, 2015; Nassar et al., 2015; Kabel et al., 2018). In humans DDP4 inhibitors administration showed decrease in PD incidence (Svenningsson et al., 2016; Brauer et al., 2020) and beneficial effect in diabetic PD patients (Jeong et al., 2021; Table 6).

The most common treatment for type 2 diabetes, metformin, showed promising results in MPTP animal models (Katila et al., 2017; Table 6). Moreover, it reduced mitochondrial respiration dysfunction, activating AMP-activated protein kinase (AMPK), which has pro-survival functions and increases α-Syn clearance in animal models of PD (Parekh et al., 2022). Recently, it has been demonstrated that metformin is able to control microglial and astrocyte activation, eventually leading to neuroprotection and controlling dyskinesia development (Ryu et al., 2020). So far, metformin treatments in humans gave rise to conflicting results (Wahlqvist et al., 2012; Ping et al., 2020).

Mitoglitazone, an antidiabetic molecule which was found to protect against MPTP toxicity in cells, rodents and nematodes, reduced the incidence of PD in diabetic patients (Brauer et al., 2015, 2020; Table 6) exerting a better effect when compared to metformin (Brakedal et al., 2017).

Furthermore, high doses of thiamine improved motor function in PD patients by acting on AGE levels (Karachalias et al., 2010; Costantini et al., 2013, 2015; Table 6).

Other molecules showed promising results in preclinical models such as, MGO-scavengers tenilsetam and aminoguanidine that reduced α-Syn aggregation while improving its clearance and motor behavior in a PD models (Vicente Miranda et al., 2017b; Table 6). Telmisartan an anti-hypertension molecule, which was shown to reduce AGEs levels in rodents, demonstrated a protective role in MPTP models (Sato et al., 2014; Table 6).

## 9. SUMOylation

The covalent addition of a small ubiquitin like modifiers (SUMO) is one of the PTM which characterizes α-Syn. SUMO is a 12 kDa protein attached covalently to the lys-residues of a protein and it is essential for normal cellular processes including cell cycle regulation, nuclear-cytosolic transport, gene transcription, protein stability, response to stress, apoptosis and many others functions (Matunis et al., 1996; Hershko and Ciechanover, 1998).

SUMOylation is mediated by a three-step reaction that involves SUMO activating enzyme (SAE1), Ubc9 conjugating enzyme and SUMO-E3 ligase (Muller et al., 2001; Wilkinson and Henley, 2010). SUMO peptides can be recycled through a process of deSUMOylation by the SUMO proteases from the Ulp/SENP family.

SUMOylation machinery and protein SUMOylation dramatically increase in response to cellular stresses, and so in PD (Zhou et al., 2004; Enserink, 2015). Furthermore, rotenone-injected mice exhibit increased α-Syn and SUMO levels (Weetman et al., 2013). SUMOylation participates in several pathways connected to PD such as regulation of DJ-1 activity, modulation of transcription factors involved in mitochondrial and lysosomal biogenesis, and regulation of mitochondrial fission machinery (Harder et al., 2004; Ariga et al., 2013; Savyon and Engelender, 2020).

SUMO has been shown to enhance the solubility of aggregation-prone proteins like α-Syn, and impaired SUMOylation increased α-Syn aggregation and toxicity in HEK293 cells and a PD rat models (Krumova et al., 2011). On the other hand, SUMOylation competes with ubiquitination on the same lys residue, protecting the protein from degradation (Rott et al., 2017; Rousseaux et al., 2018). The discrepancies seen on α-Syn aggregation may be related to the different SUMO isoforms and SUMO-ligases that may be involved in the processes (Tatham et al., 2001; Bohren et al., 2004; Wilkinson and Henley, 2010).

The only tested molecule for the interference with E1-SUMO complex formation in PD like model, is ginkgolic acid (Fukuda et al., 2009; Table 7), which decreases the levels of SUMOylation stimulating the macroautophagic clearance of α-Syn aggregates (Vijayakumaran et al., 2019).

**Table 7:** SUMOylation inhibitors tested in preclinical models of PD.

Target	Mechanism of action	Molecule name	Results from studies in preclinical models	Direct or indirect effect on α-Syn SUMOylation
E1-SUMO complex	Inhibition	Ginkgolik acid	Fukuda et al. (2009)	Direct

So far, SUMOylation targeting has been achieved especially in oncology, indeed spectomycin B1 had been proposed as therapeutic agent to cure breast cancer through the blocking of SUMOylation preventing the formation of the Ubc9-SUMO (Hirohama et al., 2013). In addition, the potent SAE inhibitor ML-792 impairs SUMO conjugation but also induces significant loss of viability in multiple cancer cell lines (He et al., 2017). On the other hand, global cellular SUMOylation is enhanced in response to interferons (Maroui et al., 2018).

## 10. Ubiquitination

The ubiquitin–proteasome system (UPS) mediates the degradation of proteins in mammalian cells (Ross and Pickart, 2004). The addition of multiple molecules of ubiquitin, a conserved 8.5-kDa polypeptide, constitute the signal for proteasome-mediated degradation. Ubiquitin–substrate ligation is mediated by different enzymatic steps which are mainly mediated by E3 ligases. These latter recognize specific substrate-based signals in a manner that is frequently regulated by covalent modification (Weissman, 2001), in which the first ubiquitin is covalently joined to proteins through an isopeptide bond between the C-terminus of ubiquitin and a lys residue, and must be proteolytically processed by ubiquitin C-terminal hydrolases (UCHs) before it can acquire activity (Weissman, 2001). Additional ubiquitins are then linked to the first one to form a polyubiquitin chain that is a potent attractive signal for the regulatory complex of the proteasome. The UPS is vitally important for protecting cells against the toxic effects of misfolded proteins (Engelender et al., 2022). The 26S proteasome consists of more than 60 subunits. It is composed by: (1) a central, barrel-shaped catalytic (20S) complex carrying multiple active sites, which are sequestered in an interior chamber that is only accessible through a narrow axial pore; (2) two distally positioned regulatory (19S) complexes which unfold the substrate polypeptide chain and translocate it through this pore and into the active-site chamber, using integral chaperone subunits placed immediately adjacent to the axial pore of the 20S complex (Ross and Pickart, 2004). Of note, studies in the post-mortem brains of sporadic PD patients showed that LB contain ubiquitinated α-Syn that is not associated with UPS impairment (Tofaris et al., 2003). However, even non-ubiquitinated α-Syn appears to be degraded by the 20S proteasome (Tofaris et al., 2001), supporting the occurrence of ubiquitin-independent mechanism of UPS-mediated α-Syn degradation in synucleinopathies.

Studies in cell models or purified systems led to conflicting results either supporting that both 20S and 26S proteasomes degrade α-Syn or failing to detect α-Syn accumulation upon UPS inhibition (Bennett et al., 1999; Tofaris et al., 2001, 2011; Webb et al., 2003; Emmanouilidou et al., 2010; Shabek et al., 2012) hinting that the UPS may play a relevant role in degrading a fraction of α-Syn, whose relative abundance may vary between cell types and experimental conditions (Stefanis et al., 2019).

Promoting the activity of the UPS can thus be considered as a possible therapeutic strategy for combating α-Syn accumulation (Engelender et al., 2022; Table 8). For instance, following evidence that p38 mitogen-activated protein kinase (MAPK) negatively regulates proteasome activity, the p38 MAPK inhibitor PD169316 has been identified as a proteasome activator that decreases α-Syn toxicity in cells (Braun et al., 2021; Engelender et al., 2022). Several p38 MAPK inhibitors tested in clinical trials for chronic inflammatory diseases and cancer may also be considered as possible UPS stimulators, though their neuroprotective effects may not be solely ascribed to UPS induction. Indeed, studies in experimental models of synucleinopathies and of other neurodegenerative diseases such as AD have shown that p38 MAPK plays a relevant role in mediating other key processes involved in neurodegeneration, neuroinflammation and disease protein-mediated brain damage (Giovannini et al., 2002, 2008; Cuenda and Rousseau, 2007).

**Table 8:** UPS modulators tested in preclinical models of PD.

Target	Mechanism of action	Molecule name	Results from studies in preclinical models	Direct or indirect on ubiquitinated or non-ubiquitinated α-Syn
p38 MAPK	Inhibition	PD169316	Braun et al. (2021)	Indirect
PKA	Activation	forskolin and analogues	Sanders and Rajagopal (2020)	Indirect
Phosphodiesterases	Inhibition	rolipram, cilostazol, vinpocetine and others	Prickaerts et al. (2017)	Indirect
20S proteasome subunit	Gate opening	chlorpromazine and some derivatives	Forster and Hill (2003), Jones et al. (2017)	Indirect
α-Syn + proteasome	α-Syn targeting to proteasome	β-synuclein-based TAT + proteasome degron petptide	Jin et al. (2021)	Direct
α-Syn	α-Syn ubiquitination and targeting to proteasome	PROTAC	Kargbo (2020)	Direct

Alternatively, compounds that work as gate-openers of the 20S proteasome by preventing the barrel closing may also promote α-Syn clearing (Forster and Hill, 2003; Jones et al., 2017). For instance, chlorpromazine and some derivatives devoid of dopamine receptors D2 binding were shown to promote the degradation of α-Syn by interacting with the 20S subunits and preventing its closure (Jones et al., 2017).

Another strategy to increase proteasomal activity is to modulate the phosphorylation status of its subunits that are influenced by several protein kinases (Kors et al., 2019). In particular, cAMP-dependent protein kinase A (PKA) phosphorylates the 19S subunits Rpt6 and Rpn6, leading to activation of 20S proteolytic activities in a process that may involve changes in proteasomal conformation (Zhang et al., 2007; Lokireddy et al., 2015). Despite the benefits of PKA activators, no positive outcome on improving cognition has been observed in clinical trials with forskolin analogs (Sanders and Rajagopal, 2020). On the other hand, several clinical trials assessing the efficacy of phosphodiesterase inhibitors are currently under way, including rolipram, cilostazol and vinpocetine (Prickaerts et al., 2017) and may hold promise for treating synucleinopathies.

A more recent approach to promote the proteasomal degradation of disease proteins is cell-penetrating peptides that specifically interact with the target protein and the proteasome. One promising peptide consists of a portion of β-synuclein peptide that interacts with α-Syn, which was fused to the cell-penetrating peptide TAT and a proteasomal degron and significantly decreased the neuronal levels of α-Syn via proteasome as well as neurotoxicity in mice (Jin et al., 2021).

Finally, the proteasomal degradation of disease proteins can also be improved Proteolysis Targeting Chimeric (PROTAC) compounds (Sakamoto et al., 2001). The technology relies on the fusion of a ligand for the target protein to a ligand for an E3 ubiquitin-ligase, such as cereblon and Van Hippel-Landau (VHL; Au et al., 2020). α-Syn-targeting PROTAC are currently in preclinical development (Kargbo, 2020).

## 11. Discussion

The evidence summarized in this review highlights the relevance of α-Syn PTMs in PD pathophysiology. In the last few years, α-Syn PTMs have been investigated as biomarker for the diagnosis and progression of PD and other synucleinopathies. Moreover, studies supporting that PTMs control structural changes in α-Syn thus influencing its aggregation propensity, have blossomed great interest for the development of innovative therapeutic strategies, that by modulating α-Syn PTM, could reduce its pathological aggregation or spreading. Interestingly, some novel therapeutic strategies modulating α-Syn PTMs are already under investigation in clinical trials. This notwithstanding, further studies are warranted to better clarify the role of PTMs on α-Syn pathophysiology, to confirm the translational potential of PTMs-modifying drugs in synucleinopathies as well as to disclose whether the evaluation of α-Syn PTMs in peripheral tissues can be a valuable readout to monitor the effect of such approaches.

## Author contributions

FL, GF, VB, and AB conceived the manuscript. VB and AB collected references, wrote the main text, and prepared illustrations and tables. AB revised manuscript text and tables. All authors contributed to the article and approved the submitted version.

## Funding

We are grateful to the Michael J. Fox Foundation for Parkinson’s Research, NY, USA (grant ID: MJFF-021179), the Multiple system atrophy coalition, USA, and the MIUR PRIN 2017-1065.

## Conflict of interest

The authors declare that the research was conducted in the absence of any commercial or financial relationships that could be construed as a potential conflict of interest.

## Publisher’s note

All claims expressed in this article are solely those of the authors and do not necessarily represent those of their affiliated organizations, or those of the publisher, the editors and the reviewers. Any product that may be evaluated in this article, or claim that may be made by its manufacturer, is not guaranteed or endorsed by the publisher.
